# Role of Small Intestine and Gut Microbiome in Plant-Based Oral Tolerance for Hemophilia

**DOI:** 10.3389/fimmu.2020.00844

**Published:** 2020-05-20

**Authors:** Sandeep R. P. Kumar, Xiaomei Wang, Nagavardhini Avuthu, Thais B. Bertolini, Cox Terhorst, Chittibabu Guda, Henry Daniell, Roland W. Herzog

**Affiliations:** ^1^Herman B Wells Center for Pediatric Research, IAPUI, Indianapolis, IN, United States; ^2^Department of Pediatrics, University of Florida, Gainesville, FL, United States; ^3^Department of Genetics, Cell Biology and Anatomy, University of Nebraska Medical Center, Omaha, NE, United States; ^4^Division of Immunology, Beth Israel Deaconess Medical Center (BIDMC), Harvard Medical School, Boston, MA, United States; ^5^Department of Basic and Translational Sciences, School of Dental Medicine, University of Pennsylvania, Philadelphia, PA, United States

**Keywords:** hemophilia, oral tolerance, transgenic plant, regulatory T (Treg) cell, factor IX

## Abstract

Fusion proteins, which consist of factor VIII or factor IX and the transmucosal carrier cholera toxin subunit B, expressed in chloroplasts and bioencapsulated within plant cells, initiate tolerogenic immune responses in the intestine when administered orally. This approach induces regulatory T cells (Treg), which suppress inhibitory antibody formation directed at hemophilia proteins induced by intravenous replacement therapy in hemophilia A and B mice. Further analyses of Treg CD4^+^ lymphocyte sub-populations in hemophilia B mice reveal a marked increase in the frequency of CD4^+^CD25^−^FoxP3^−^LAP^+^ T cells (but not of CD4^+^CD25^+^FoxP3^+^ T cells) in the lamina propria of the small but not large intestine. The adoptive transfer of very small numbers of CD4^+^CD25^−^LAP^+^ Treg isolated from the spleen of tolerized mice was superior in suppression of antibodies directed against FIX when compared to CD4^+^CD25^+^ T cells. Thus, tolerance induction by oral delivery of antigens bioencapsulated in plant cells occurs via the unique immune system of the small intestine, and suppression of antibody formation is primarily carried out by induced latency-associated peptide (LAP) expressing Treg that likely migrate to the spleen. Tolerogenic antigen presentation in the small intestine requires partial enzymatic degradation of plant cell wall by commensal bacteria in order to release the antigen. Microbiome analysis of hemophilia B mice showed marked differences between small and large intestine. Remarkably, bacterial species known to produce a broad spectrum of enzymes involved in degradation of plant cell wall components were found in the small intestine, in particular in the duodenum. These were highly distinct from populations of cell wall degrading bacteria found in the large intestine. Therefore, FIX antigen presentation and Treg induction by the immune system of the small intestine relies on activity of a distinct microbiome that can potentially be augmented to further enhance this approach.

## Introduction

The incidence of hemophilia is ~1 in 5,000 male births worldwide. Mutations in either the serine protease factor IX (FIX) or its co-factor, factor VIII (FVIII) that reduce coagulation activity to <1% of normal typically result in severe disease, which is characterized by frequent and potentially life-threatening bleeds. Deficiency of FVIII is referred to as hemophilia A, while FIX deficiency is called hemophilia B. Bleeding can be prevented by frequent intravenous injections of recombinant or plasma-derived factor product (2–3 times per week). However, 20–30% of severe hemophilia A patients develop neutralizing antibodies that inhibit coagulation activity and are therefore referred to as “inhibitors.”

Antibody formation directed at the newly-introduced proteins represents a serious complication in protein replacement therapy for the X-linked bleeding disorder hemophilia (1–5). One partial remedy is ITI (“immune tolerance induction,” consisting of daily high-dose intravenous infusion of FVIII), which however may take months to years and can cost >$1M (1). Incidence of inhibitor formation against FIX is lower in treatment of hemophilia B (estimated ~5% of patients). However, up to 50% of patients with FIX inhibitors experience anaphylactic reactions and/or nephrotic syndrome upon further exposure to FIX (4).

Bypassing agents are available to restore hemostasis, but these are very expensive and have to be more carefully dosed to avoid thrombosis. More recently, a bispecific antibody has been developed that mimics FVIII and promotes coagulation in hemophilia A patients even in the presence of an inhibitor (6, 7). No such therapy is available for hemophilia B, although progress has been made in development of an RNAi therapy that down-regulates expression of the anti-coagulant protein anti-thrombin III, thereby promoting coagulation in hemophilia A or B patients (8). However, none of these bypassing therapies completely restore hemostasis nor do they induce tolerance.

Hence, we and others have been developing diverse novel protocols aimed at reversal of inhibitor formation by immune tolerance induction (ITI), which have primarily been tested in hemophilic mice in which either the *F8* or *F9* gene had been deleted (9–14). These studies employ a range of strategies, including lymphocyte-based therapies and administration of small molecule/protein/antibody drugs, which modulate distinct immune responses (5). However, methodologies that allow for a prediction of inhibitor formation by individual patients need to be improved and a better understanding of risk factors will be requisite.

We are currently evaluating an alternative approach, which employs introduction of the coagulation factor antigen through a tolerogenic route without the use of immune suppressive drugs or genetic engineering. To this end, we have developed a plant cell-based oral tolerance approach (15–21). FVIII and FIX antigens have been expressed in chloroplast transgenic (transplastomic) crop plants for high levels of antigen production in green leaves. Initially developed in tobacco, this platform has now been optimized in the edible crop plant lettuce, thereby moving closer to clinical application (16, 18, 20, 22). While early studies expressed the native human genes, subsequence studies employed codon optimization to increase antigen expression 10–50-fold in chloroplasts (18). Plants can be grown under soil-free conditions, and leaves harvested and freeze-dried and ultimately converted to a dry powder. This cost-effective production system does not require extraction and purification of the antigen. In fact, antigens are stable in lyophilized plant cells for 2–3 years when stored at ambient temperature (16, 20, 23). Commercial scale production in cGMP hydroponic facility has been demonstrated for several human blood proteins (16, 20, 24). Most importantly, methods have been developed to remove antibiotic resistance genes from chloroplast genomes of edible plant cells producing enzymes or biopharmaceuticals (20, 24, 25). Plant cell wall protects antigens from acid and enzymes in the stomach because they do not cleave Beta1–4, 1–6 linkages in plant cell wall polymers (17, 26). However, commensal bacteria release plant cell wall degrading enzymes thereby releasing antigens in the gut lumen (17, 24). Moreover, antigens are expressed as fusion proteins between the coagulation factor and a transmucosal carrier. N-terminal fusion of CTB (cholera toxin B subunit, an FDA approved antigen), results in pentamer formation and, upon release in the intestine, binding to GM receptor on gut epithelial cells and transmucosal delivery to the immune system (13, 19, 27–29). A furin cleavage site has been engineered between CTB and the antigen so the antigen is released, while CTB is retained in cells that have taken up the fusion protein (30). A major advantage of targeted delivery is efficacy at low antigen doses (18, 20, 21).

Repeated oral delivery of plant cells expressing CTB-fused antigen has been effective in suppression of inhibitor formation against FVIII in hemophilia A mice and against FIX in hemophilia B mice and dogs that were subsequently treated with intravenous FVIII or FIX therapy (18–21). Moreover, IgE formation and thus anaphylaxis against FIX was prevented in hemophilia B mice and dogs (13, 16, 20, 21). Studies in hemophilia B mice revealed a complex mechanism of tolerance induction that involves changes in subsets of dendritic cell (DCs) and regulatory T cell (Treg) populations (13, 15, 19). Here, we demonstrate induction of CD4^+^CD25^−^FoxP3^−^LAP^+^ Treg (“LAP^+^ Treg”) in the small but not large intestine of hemophilia B mice and further support their role in suppressing antibody formation.

## Materials and Methods

### Animal Experiments and Adoptive T Cell Transfer

Hemophilia B mice with targeted *F9* gene deletion on C3H/HeJ background were as published (20, 21, 31). Male mice 6–8 weeks of age were used for the experiments and housed under special pathogen-free conditions. For oral tolerance induction, freeze-dried powder of lettuce (*Lactuca sativa*) cv. Simpson Elite with CTB-FIX transgene integrated into chloroplast genome (homoplasmic transplastomic plants) were prepared as published (16, 20). The transgene expresses cholera toxin B subunit (CTB) fused to the N terminus of the mature form of human coagulation factor IX (FIX). Plant material (containing 5 μg CTB-FIX antigen per dose suspended in 200 μl of sterile PBS) was orally delivered via gavage twice per week for 2 months using a 20-G bulb-tipped gastric gavage needle. For intravenous challenge with human FIX antigen, mice were administrated 1 IU FIX (Benefix, Pfizer, New York, NY) into the tail vein once per week for 2 months, starting 4 weeks after initiation of the oral tolerance regimen. Control groups received only intravenous FIX but no gavages. Subsequently, splenocyte preparations were pooled for each experimental group (*n* = 10 per group), and CD4^+^ T cells were isolated by magnetic sorting using the CD4^+^ T cell isolation kits from Miltenyi Biotech (Auburn, CA). Isolated CD4^+^ T cells were stained with anti-mouse CD4-eflour 450 (clone RM4–5, eBioscience, San Diego, CA), LAP-APC (clone TW7–16B4, BioLegend, San Diego, CA) and CD25-PE (clone PC61, BD Biosciences, San Jose, CA) antibodies. Flow sorting was employed to purify CD4^+^CD25^+^ T cells and CD4^+^CD25^−^LAP^+^ T cells using FACS Aria II cell-sorter (BD Biosciences, San Jose, CA). Post-sort analysis of cells confirmed more than 95% purity. Purified live cells (as determined by trypan blue staining) were adoptively transferred into naive strain-matched mice via tail vein injection (300,000 cells/mouse). After 24 h, recipient mice were immunized by subcutaneous injection of 1 IU FIX formulated in Sigma Adjuvant System. Blood samples were collected 3 weeks later, and plasma levels of FIX-specific immunoglobulins were measured by ELISA. Statistical analysis was performed using unpaired student *t*-test.

### Isolation of Gut Lymphocytes

Mice were tolerized as explained above, and small intestinal (ileum and distal jejunum) and large intestinal (colon) tissues were harvested, homogenized, and treated with RBC lysis buffer (Sigma, St. Louis, MO). Intestine was placed in and flushed with ice-cold PBS. Attached tissues such as fat tissue or Peyer's patches were removed, and the intestine cut longitudinally. Tissue was further cut into small pieces (<0.5 cm) and washed multiple times with ice-cold PBS. For pre-digestion, tissue was transferred into 50 ml tube, containing 20 ml pre-digestion solution (pre-heated to 37°C): 1 × HBSS (w/o Ca^2+^, or Mg^2+^, or phenol red), 10 mM HEPES, 5 mM EDTA, 1 mM DTT, and 5% FCS (fetal calf serum). Tissue was then incubated for 20 min at 37°C while slowly rotating horizontally. After brief vortex (10 s), tissue was passed through a 100-μm cell strainer (without application of pressure). Incubation and cell straining were repeated once. All flow throughs were collected and subsequently combined to isolate intraepithelial lymphocytes (IELs), while the cells retained by the strainer were combined to isolate lamina propria lymphocytes (LPLs).

For LPLs isolation, cells were placed into pre-warmed buffer of the following composition: 1 × HBSS (w/o Ca^2+^, or Mg^2+^, or phenol red), 10 mM HEPES, 0.5 mg/ml collagenase D, 0.5 mg/ml DNase, and 5% FCS. Incubation was again performed at 37°C with horizontal tube rotation, this time for 30 min. Cells were passed through 100-μm cell strainer, followed by addition of excess FACS buffer. Cells were collected by centrifugation for 10 min at 300 g, followed by resuspension in FACS buffer and storage on ice. Finally, LPL or IEL fractions were each purified by Percoll gradient centrifugation (40:80%, 20 min, 1,000 g, room temperature). Cells were recovered, washed in FACS buffer, and ultimately resuspended in FACS buffer for fluorescent antibody staining and flow cytometry.

### Flow Cytometry

Analysis by flow cytometry was performed as published (13). Surface staining with antibodies was performed at 4°C for 30 min in PBS, followed by addition of viability dye eFluor 506 (or APC-Cy7) at 4°C for 30 min in PBS. Fixation and Foxp3 Alexa Fluor 647 stain was performed with the transcriptional factor staining buffer set from eBiosciences (San Diego, CA). Isotype control, single positive, and unstained cells served as controls. Flow cytometry was performed using the LSR II system (BD Bioscience, San Jose, CA), and data were analyzed with FlowJo (BD Life Sciences, Franklin Lake, NJ) or FCSExpress software (*De Novo* Software, Los Angeles, CA). Antibodies against murine antigens were obtained from eBiosciences and included anti-CD4 (eFluor450 conjugated), -CD25 (PE), -LAP (PerCP-eFluor710), and -FoxP3 (Alexa Fluor 647).

### Microbiome Analysis

Gut content from duodenum, jejunum, and ileum segments of small intestine as well as large intestine were collected, and DNA was extracted using QIAamp PowerFecal Pro DNA Kit from Qiagen (Hilden, Germany) according to the manufacturer's protocols. To assess the microbial diversity of duodenum, jejunum/ileum and large intestine, the *QIAseq* 16S/ITS libraries were developed for nine variable regions of 16S rRNA (six amplicons covering v1v2, v2v3, v3v4, v4v5, v5v7, and v7v9 regions) and eukaryote ITS (internal transcribed spacer) using QIAseq 16S/ITS screening panel from the extracted DNA. Sequencing libraries were labeled with different multiplex indexing barcodes (Table 1) for each sample. The indexed libraries were quantified and paired-end (2 × 251 bp) *MiSeq* sequencing was performed at the Center for Medical Genomics at Indiana University School of Medicine, using reagents from Qiagen. Raw sequencing reads were demultiplexed into separate FASTQ files for each sample with reads from each variable region. Sequence quality of the demultiplexed FASTQ files were assessed using *FASTQC* tool and imported into *QIIME2* environment. Sequencing data were analyzed using *QIIME2 2019.10* pipeline to obtain the microbial diversity and abundance in the duodenum, jejunum, and large intestine regions (32). The reads from all the samples were separated based on amplified variable regions (v1v2, v2v3, v3v4, v4v5, v5v7, v7v9, and ITS) using a QIIME2 *cutadapt* plugin. *QIIME2* with *DADA2* denoising method was used for quality control and to identify amplicon sequence variants (ASVs) based on amplified variable regions. The parameters “–p-trunc-len” and “–p-trim-left” in *QIIME2 DADA2 denoise-paired* plugin were altered based on read quality distribution. The *QIIME2 Naïve Bayes* classifiers were built from *SILVA 132* 99% OTUs specifically for primer sets used for amplification of variable regions (33). Then the *QIIME2 q2-feature-classifier* plugin was used for taxonomic assignments of ASVs using the default settings. For ITS-based classification of eukaryotic species, *QIIME2* classifier based on *UNITE* database was used (34), however as we found a small number of taxa only from Protista, the eukaryotic analysis was not advanced to the next level. The identified taxonomy tables were filtered for rare and unclassified ASVs. The taxonomic composition based on each variable region was compared and viewed using *QIIME2 taxa barplot* plugin at each taxonomic level from kingdom down to species. Alpha diversity analysis was performed using QIIME2 q2-diversity plugin. The enzyme producing potentials of microbes in duodenum, jejunum, and large intestine regions were identified using *PICRUSt2 v2.2* algorithm based on the sequences and abundance profiles of the ASVs identified with QIIME2 (35). Predicted microbial enzymes were mapped to microbes at various taxonomic levels. The abundance of enzymes involved in the plant cell wall degradation were compared among duodenum, jejunum, and large intestine regions using *STAMP 2.1.3* software (36). Because different amplicon regions show different microbial compositions and consequently different potentials for producing the enzymes of interest, the highest abundance of each enzyme in any amplicon region was used for plotting the enzyme box plots across all three regions.

**Table 1:** Samples used for microbiome analysis and its barcodes.

**Sample-id**	**Barcode-sequence**	**Body site**
1_Duo_DNA	ATTACTCG+TATAGCCT	Duodenum
2_Duo_DNA	CGCTCATT+CCTATCCT	Duodenum
3_Duo_DNA	ATTCAGAA+TATAGCCT	Duodenum
4_duo_DNA	TCCGGAGA+GGCTCTGA	Duodenum
1_jej_DNA	ATTCAGAA+CCTATCCT	Jejunum
2_jej_DNA	GAATTCGT+GGCTCTGA	Jejunum
3_jej_DNA	GAATTCGT+ATAGAGGC	Jejunum
4_jej_DNA	CGCTCATT+TATAGCCT	Jejunum
1_Large_DNA	TCCGGAGA+ATAGAGGC	Large Intestine
2_large_DNA	GAGATTCC+GGCTCTGA	Large Intestine
3_large_DNA	ATTACTCG+CCTATCCT	Large Intestine
4_large_DNA	GAGATTCC+ATAGAGGC	Large Intestine

## Results

### LAP^+^CD25^−^ but Not CD4^+^CD25^+^ T Splenocytes Suppress Anti-FIX Formation After Adoptive Transfer of Low Cell Numbers

**Figure 1:**
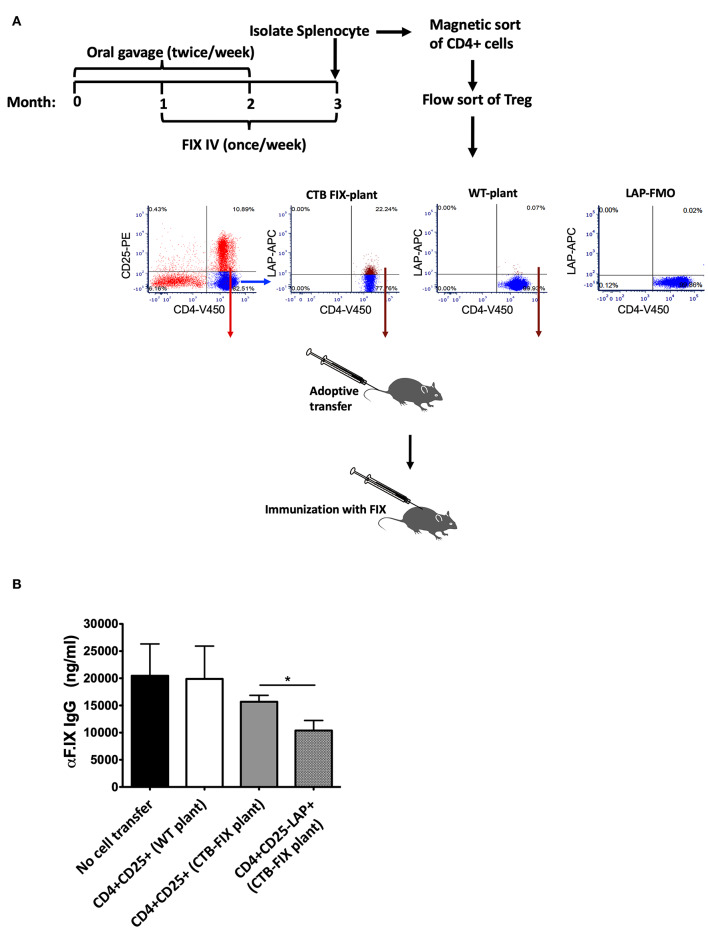
Suppression of antibody formation against FIX after adoptive transfer of Treg. **(A)** Experimental outline of oral tolerance with bioencapsulated CTB-FIX/intravenous treatment with FIX, T cell isolation, adoptive transfer, and immunization. Donor hemophilia B mice had been treated in this manner or were naïve control mice. Flow cytometry plots show results from purification of splenic CD4^+^CD25^+^ T cells and CD4^+^CD25^−^LAP^+^ T cells by flow sorting. **(B)** FIX-specific IgG titers in mice that received either CD4^+^CD25^+^ T cells or CD4^+^CD25^−^LAP^+^ T cells after immunization with FIX. Results are average ±SD (*n* = 5/group). * indicates *P* < 0.05.

During oral tolerance induction, total CD4^+^CD25^+^FoxP3^+^ Treg frequencies do not significantly change in various organs examined, which is in contrast to the increase in frequencies of LAP^+^ Treg. Nonetheless, we found suppression of antibody formation against FIX to be equally potent as with CD4^+^CD25^−^LAP^+^ following adoptive transfer (while non-CD4^+^ cells or CD4^+^CD25^−^LAP^−^ failed to suppress) (13). In these studies, we had transferred 1 × 10^6^ cells of either subset from tolerized to recipient mice. Here, we transferred more limited cell numbers (300,000 per recipient mouse) from hemophilia B mice that had been orally tolerized to FIX to naive mice of the same strain (Figure 1A). Recipient mice were challenged with FIX in adjuvant, followed by measurement of antibody titers 3 weeks later. Antibody titers were similar for mice that received CD4^+^CD25^+^ T cells isolated from the spleens of orally tolerized or untreated control mice (*n* = 5/experimental recipient group, Figure 1B). In contrast, CD4^+^CD25^−^LAP^+^ T cells from orally tolerized animals suppressed antibody formation, with titers significantly lower as compared to after CD4^+^CD25^+^ T cells transfer (Figure 1B). Therefore, LAP^+^ Treg are the main source of systemic suppression of antibody formation. These cells are induced by antigen administration on mucosal interphases, and hence we were unable to obtain sufficient numbers of CD4^+^CD25^−^LAP^+^ T cells in unfed control mice as illustrated in Figure 1A.

### LAP^+^ Treg Are Expanded in the Lamina Propria of the Small but Not the Large Intestine

**Figure 2:**
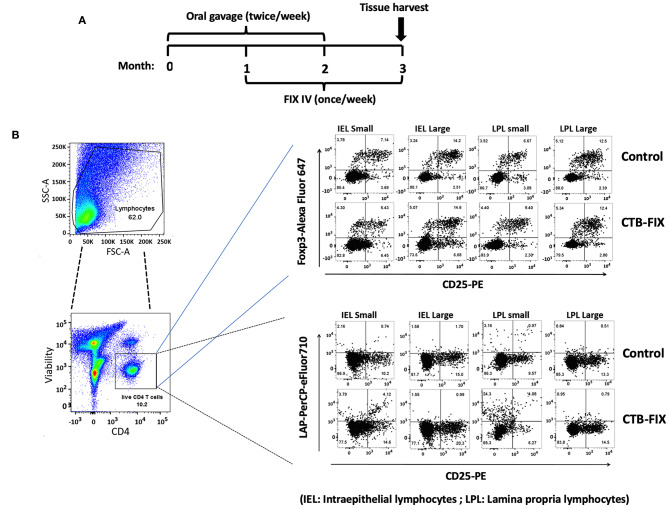
Analysis of Treg subsets in intraepithelial lymphocyte (IEL) and lamina propria lymphocyte (LPL) populations of small and large intestine of hemophilia B mice using flow cytometry. **(A)** Time course of oral tolerance regimen with CTB-FIX expressing plant cells and treatment by intravenous injection of recombinant FIX protein. **(B)** Gating scheme and examples of flow cytometry results. Examples of small intestine (“small”) and large intestine (“large”) results are shown for CD4^+^CD25^+^FoxP3^+^ T cells and CD4^+^CD25^−^LAP^+^ T cells. Control mice had not received any treatment.

**Figure 3:**
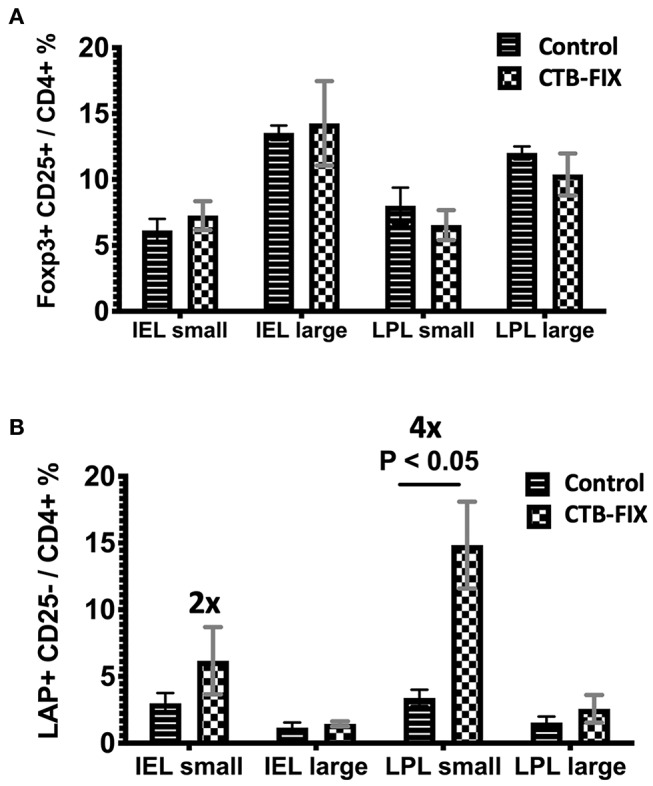
Frequencies of **(A)** CD4^+^CD25^+^FoxP3^+^ T cells and **(B)** CD4^+^CD25^−^LAP^+^ T cells among IEL and LPL populations of small and large intestines of orally tolerized and naïve control hemophilia B mice. Results are average ±SD (*n* = 5/group).

Next, we examined the relative frequencies of FoxP3^+^ and LAP^+^ Treg in the immune system lining the small and large intestines in hemophilia B mice upon completion of our oral tolerance regimen (Figure 2 and Supplementary Figure S1). Consistent with our prior findings (13, 19), no increases in total frequencies of FoxP3^+^ Treg were found in intraepithelial lymphocytes (IEL) or lamina propria lymphocytes (LPL) of either organ (Figure 3A). Compared to untreated mice, frequencies of LAP^+^ Treg also did not change in the large intestine (*n* = 5 per group). However, LAP^+^ Treg frequencies were significantly increased (by 4-fold, Figure 3B) among LPLs of the small intestine (a more modest 2-fold, non-significant increase was seen in IPLs of the small intestine, Figure 3B). Therefore, our results further support the prevailing model that oral tolerance induction primarily occurs in the immune system of the small intestine.

### Microbiome Analysis Reveals Presence of Bacteria in Duodenum That Are Capable of Producing Plant Cell Wall Degrading Enzymes

**Figure 4:**
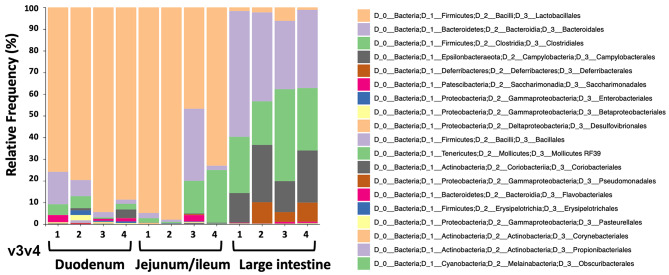
Relative frequencies of bacterial orders in duodenum, jejunum/ileum, and small intestine of hemophilia B mice (*n* = 4) as determined by bioinformatic analysis of NGS data. The example shown here is for data obtained from amplification of variable region v3v4.

To study the microbiome of the hemophilia B mice, intestinal contents of four C3H/HeJ F9^−/−^ mice of the same colony used in the oral tolerance experiments were collected for DNA extraction. Contents of duodenum and jejunum/ileum portions of the small intestine were collected separately, in addition to contents from the large intestine (colon). All the nine variable regions of the 16S ribosomal RNA gene covered by six amplicons were analyzed by next-generation sequencing (NGS). Consistent with findings by others (37), the microbiomes of the duodenum and jejunum/ileum portion of the small intestine were dominated by bacteria of the *Lactobacillales* order (Figure 4, Supplementary Figures S2A–E, and Table 1). The microbiome of the large intestine was distinct from the small intestine with more alpha diversity (Supplementary Figures S3A,B), and consisted predominantly of bacteria of the *Bacteroidales, Clostridiales, Campylobacterales*, and *Deferribacterales* orders. Although both locations contain considerable populations of *Firmicutes*, these are predominantly *Lactobacillales* in the small intestine and *Clostridiales* in the large intestine. Also, bacteria from phylum, *Bacteriodetes* are significantly higher in the large intestine compared to the small intestine.

**Figure 5:**
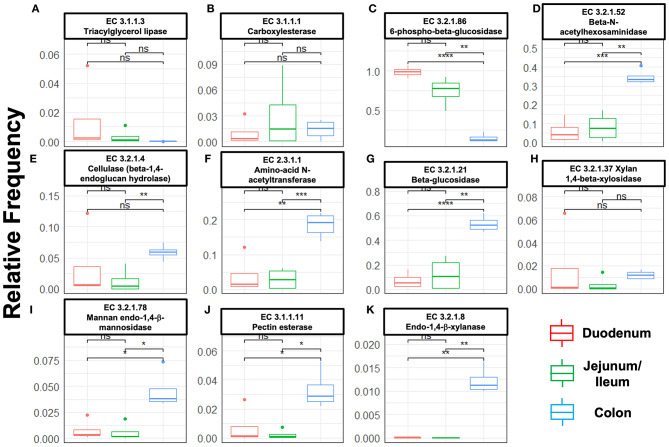
Species relative frequencies of bacteria producing the following enzymes in duodenum, jejunum/ileum, and large intestine of hemophilia B mice as predicted by PICRUSt2 on different 16S rRNA variable region taxonomic profiles. Results are shown for six variable regions analyzed by NGS (Statistical method *t*-test was used, asterisks represent significance level of a pairwise *t*-test with *P*-values i.e., ‘****' ≤ 1e-04, ‘***' ≤ 0.001, ‘**' ≤ 0.01, ‘*' ≤ 0.05, ns > 0.05 (not significant). **(A)** Triacylglycerol lipase. **(B)** Carboxylesterase. **(C)** 6-phospho-β-glucosidase. **(D)** β-N-acetylhexosaminidase. **(E)** Cellulase (β-1,4-endoglucan hydrolase). **(F)** Amino-acid N-acetyltransferase. **(G)** β-glucosidase. **(H)** Xylan 1,4-β-xylosidase. **(I)** Mannan endo-1,4-β-mannosidase. **(J)** Pectinesterase. **(K)** Endo-1,4-β-xylanase.

**Figure 6:**
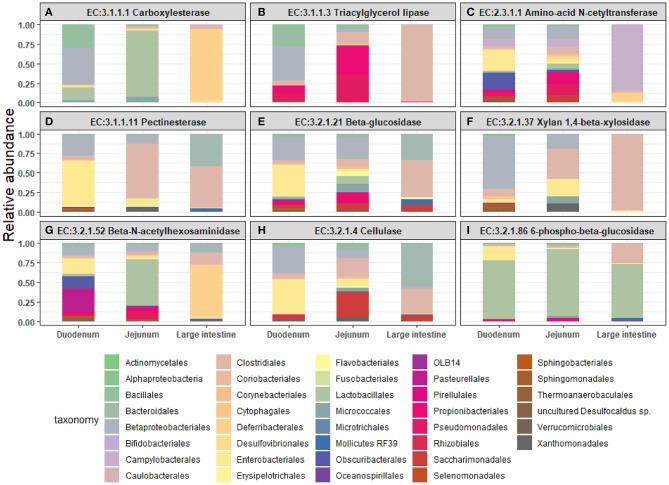
Relative abundance of bacterial orders producing enzymes that degrade plant cell wall components were identified in the duodenum, jejunum, and large intestine of hemophilia B mice. Results are shown as highest abundance of each enzyme from the tested amplicon regions in duodenum, jejunum/ileum, and large intestine of the hemophilia B mice. **(A)** Carboxylesterase. **(B)** Triacylglycerol lipase. **(C)** Amino-acid N-acetyltransferase. **(D)** Pectinesterase. **(E)** β-glucosidase. **(F)** Xylan 1,4-β-xylosidase. **(G)** β-N-acetylhexosaminidase. **(H)** Cellulase (β-1,4-endoglucan hydrolase). **(I)** 6-phospho-β-glucosidase.

Next, we mined the data for frequencies of species that are known to produce enzymes involved in degradation of plant cell wall components such as cellulose, xylan, mannan, and pectin, or in degradation of lipids or generation of glucose (Table 2 and Supplementary Figure S4). As expected, the large intestine provided higher frequencies of bacteria producing β-N-acetylhexosaminidase [EC 3.2.1.52], cellulase (β-1,4-endoglucanase) [EC 3.2.1.4], amino-acid N-acetyltransferase [EC 2.3.1.1], β-glucosidase [EC 3.2.1.21], mannan endo-1,4-β-mannosidase [EC 3.2.1.78], and pectinesterase [EC 3.1.1.11] (Figures 5D–G,I–K) compared to duodenum and jejunum/ileum because of its higher microbial diversity. Producers of endo-1,4-β-xylanase [EC 3.2.1.8] were only detected in the large intestine (Figure 5K). The jejunum/ileum section only contained higher levels of bacteria producing 6-phospho-β-glucosidase [EC 3.2.1.86], while producers of carboxylesterase [EC 3.1.1.1] were similarly abundant as for large intestine (Figure 5C). Especially, the duodenum population was dominated by producers of 6-phospho-β-glucosidase [EC 3.2.1.86] and higher levels of triacylglycerol lipase [EC 3.1.1.3] than other locations (Figures 5A,C). Interestingly, there was also strong evidence for cellulase, β-N-acetylhexosaminidase, amino-acid N-acetyltransferase, β-glucosidase, xylan 1,4-β-xylosidase, and pectinesterase producers in the small intestine region (Figures 5B,D–H,J). Although differences between duodenum and ileum/jenunum did not reach statistical significance, the duodenum of the hemophilia B mice trended to show higher frequencies of bacteria producing enzymes for breakdown of multiple cell wall components (Figures 5A,C,E,H,J and Supplementary Table S1). Comparison of the relative contribution of different families of bacteria to the production of these particular enzymes in different segments of the gut revealed that these populations are highly distinct between the duodenum, jejunum/ileum, and the large intestine (Figures 6A–H). For example, carboxylesterase [EC 3.1.1.1] producers are dominated by *Defferibacteracea* in the large intestine and *Lactobacillaceae* in the jejunum/ileum, while three different families (*Burkholderiaceae, Lactobacillaceae*, and *Staphylococcaceae*) contribute similarly in the duodenum (Figure 6A). Another example is the high contribution of *Flavobacteriaceae* to cellulase production in the duodenum, while lacking contribution of *Clostridiales* that is seen in the colon (Figure 5H). The exception was 6-phospho-β-glucosidase [EC 3.2.1.86], which appears to be predominantly produced by *Lactobacillaceae* in all segments (Figure 6I).

**Table 2:** Plant cell wall degrading bacterial enzymes produced by species identified in microbiome of hemophilia B mice.

EC 2.3.1.1	Amino-acid N-acetyltransferase
EC 3.2.1.4	Cellulase (endoglucanase/β-endoglucan hydrolase)
EC 3.2.1.78	Mannan endo-1,4-β-mannosidase
EC 3.2.1.21	β-glucosidase
EC 3.2.1.37	Xylan 1,4-β-xylosidase
EC 3.2.1.52	β-N-acetylhexosaminidase
EC 3.2.1.86	6-phospho-β-glucosidase
EC 3.1.1.1	Carboxylesterase
EC 3.1.1.3	Triacylglycerol lipase
EC 3.2.1.8	Endo-1,4-β-xylanase
EC 3.1.1.11	Pectinesterase

## Discussion

In our prior studies, up-take of FIX antigen by epithelial cells and DCs (including CD103^+^ DCs, which are critical for Treg induction) was observed in all portions of the small intestine (13). Although the immune system of the colon contains large numbers of CD4^+^CD25^+^FoxP3^+^ Treg (“FoxP3^+^ Treg”; in part to prevent inflammatory responses to the large bacterial population), oral tolerance induction is believed to be a function of the small intestine's immune system (38–41). Among the various subsets of Treg, our protocol most robustly induces CD4^+^CD25^−^LAP^+^ Treg, whose frequencies are substantially increased in Peyer's patches and mesenteric lymph nodes (MLNs), while still showing significant increases in spleens (13, 19), which is further supported by our new data. Additionally, we now demonstrate that plant cell-based oral tolerance induces LAP^+^ Treg in the mucosal immune system of the small intestine only, thus further supporting its crucial role in this approach. Detection of LAP (latency-associated peptide of TGF-β) on the surface of T cells reflects high expression of TGF-β, a cytokine that is expressed by CD103^+^ DCs and that is required for induction of FoxP3^+^ Treg and LAP^+^ Treg (42–44). The latter suppress in a TGF-β dependent manner and may serve as a biomarker for oral tolerance induction (18).

Antigen-specific Treg induction in the small intestine requires antigen release from the plant cells. Bioencapsulated antigens are protected from acid hydrolysis and digestive enzymes in the stomach through the β-1,4 and β-1,6 linkages of plant cell wall components that mammalian enzymes cannot hydrolyze. Therefore, enzymatic activities provided by the microbiome of the small intestine are critical to release FVIII or FIX antigens for delivery to the immune system and Treg induction. The microbiome of the small intestine is distinct from that of the large intestine and dominated by *Firmicutes* that are of the order *Lactobacillales*. This finding is consistent with the greater frequency of producers of 6-phospho-β-glucosidase as compared to the large intestine, highlighting the role of the small intestine in nutrient absorption. The large intestine has overall greater microbial diversity but also contains a large proportion of *Firmicutes*. These are however mostly *Clostridiales*, which are known to induce FoxP3^+^ Treg in the colon (45). Less is known about the role of the small intestine's microbiome in Treg induction, a function that is critical for oral tolerance.

In order to begin to define the role of the small intestine's microbiome in Treg induction in plant cell-based oral tolerance, we reasoned that we needed to first address the question of antigen release. Plant cell wall degrading microbes are mostly studied for the colon, addressing end digestion rather than nutrient absorption and oral immune tolerance, which are functions of the small intestine. Here, we find that the microbiome of the small intestine, while not as capable as the large intestine, does provide diversity of enzyme producers that degrade various components of the plant cell wall and greater levels of triacylglycerol lipase and 6-phospho-β-glucosidase producers. Within the small intestine, the duodenum tended to have a greater capacity and diversity of such enzyme producers compared to the jejunum/ileum. It should also be noted that not all enzymes required for complete cell wall degradation are needed for antigen release which merely relies on sufficient disruption of the cell wall integrity. The duodenum likely functions as an initial place for breakdown of plant cells, so that antigens can be released for tolerance induction. The jejunum/ileum location contains Peyer's patches and has important functions in antigen uptake and processing (39, 41).

Interestingly, composition of the bacterial population that produces the diverse cell wall producing enzymes is highly distinct between the duodenum and the large intestine. This feature could potentially be exploited to design probiotics for duodenum-specific delivery of enzyme producers. For example, with the exception of 6-phospho-β-glucosidase, the enzymatic activities that we investigated here are provided by bacteria that do not belong to the order of *Lactobacillales*, which are by far the most abundant order in the small intestine. Instead, the enzymatic activities are contributed by other types of bacteria that are present but infrequent in the small intestine, such as *Burkholderiales* or *Flavobacteriales*. Therefore, Daniell et al. have recently generated plants expressing cell wall degrading enzymes (24, 25). Importantly, oral delivery of pectinase, endoglucanase, exoglucanase, and mannanase before feeding plants cells expressing therapeutic proteins almost doubled drug levels in plasma (46). This method would provide more precise dosing of the required enzymatic activity than a probiotic approach.

While not further investigated here, we previously also found evidence for induction of Tr1 (type 1 regulatory T) cells in the lamina propria of orally tolerized mice (13). These cells are known to produce large amounts of IL-10. FoxP3^+^ Treg on mucosal interphases and LAP^+^ Treg also produce IL-10, and our oral tolerance protocol was unsuccessful in hemophilia B mice deficient in IL-10 (13). Even though our studies on other tolerance induction protocols showed that IL-10 is not generally required for Treg induction or their ability to suppress antibody formation, IL-10 is critical for plant cell-based oral tolerance induction (13, 47, 48). Moreover, we found that IL-10 and TGF-β is mainly expressed by LAP^+^ Treg upon antigen re-stimulation of splenocytes from orally tolerized mice. Although our previous adoptive transfer studies do not entirely rule out that contamination with small numbers of LAP^+^ Treg contributed to suppression by CD4^+^CD25^+^ T cells, the bulk of the literature on oral tolerance to food antigens supports our interpretation that FoxP3^+^ Treg contribute to oral tolerance induction to FVIII and FIX in our studies (38, 40, 49). It has been proposed that FoxP3^+^ Treg induced in the MLN by CD103^+^ DCs (following antigen uptake in the LP and migration to the MLN) subsequently migrate to the LP, where additional stimulation with antigen results in their further expansion (49). Through expression of IL-10 and other molecules, FoxP3^+^ Treg and Tr1 cells may help shape a local environment that supports induction of LAP^+^ Treg, which are most robustly induced and thus constitute the main type of Treg that suppresses systemic antibody/inhibitor formation against FVIII or FIX in plant-based oral tolerance. Certain species of *Clostridium* and *Bacteriodes* have been shown to induce IL-10 producing FoxP3^+^ Treg, e.g., via certain polysaccharides (50). Such effects have not yet been documented for other subsets of Treg such as LAP^+^ Treg or Tr1 cells. *Clostridium* species are controlling FoxP3^+^ Treg and Th17 differentiation and expansion, e.g., through production of short chain fatty acids (SCFAs) (50–52), and elimination of FoxP3^+^ Treg can lead to increased populations of *Firmicutes* (53). Similar to FoxP3^+^ Treg and Th17 cells, induction of LAP^+^ Treg depends on TGF-β, and LAP^+^ Treg are major contributors of IL-10 production in our plant cell-based induced immune regulation of responses against FVIII or FIX (13). One would therefore expect similar effects of the microbiome on LAP^+^ Treg induction. However, the findings summarized above are based on observations on the colonic microbiome and colonic Treg, while LAP^+^ Treg are induced in the mucosa of the small intestine. Analogous mechanisms in the small intestine that may impact oral tolerance remain to be discovered.

In conclusion, suppression of antibody formation by oral tolerance by administration of CTB-FIX bioencapsulated in plant cells is primarily performed by LAP^+^ Treg, which are expanded in the immune system of the small but not the large intestine. Therefore, optimal release of antigen from the plant cells in the small intestine and delivery to the associated immune system is key for success of this strategy. Bacteria capable of providing the required enzymatic activities are present in the small intestine (in particular in the duodenum). The composition of this population is distinct from that of the large intestine, and their augmentation may further enhance plant cell-based oral tolerance induction.

## Data Availability Statement

All datasets generated for this study are included in the article/Supplementary Material.

## Ethics Statement

All animals are maintained under specific pathogen-free conditions at the University of Florida animal facility. Experiments were performed according to the guidelines of the Institutional Animal Care and Use Committee (IACUC) at the University of Florida.

## Author Contributions

SK, XW, and TB performed experiments. SK, XW, NA, CT, CG, HD, and RH designed, analyzed, and interpreted experiments. SK, NA, TB, CT, CG, HD, and RH wrote the manuscript. RH supervised the study.

## Conflict of Interest

HD and RH have received funds from Takeda Therapeutics to further develop oral tolerance to factor VIII. RH has received funds from Luye R&D (Boston) to develop gene therapy for hemophilia. The remaining authors declare that the research was conducted in the absence of any commercial or financial relationships that could be construed as a potential conflict of interest. The reviewer HW declared a shared affiliation, with no collaboration, with one of the authors, CT, to the handling editor at the time of review.
