# Supplementary material for: Role of Small Intestine and Gut Microbiome in Plant-Based Oral Tolerance for Hemophilia
Source: Front Immunol. 2020 May 20;11:844. doi: 10.3389/fimmu.2020.00844 (PMC7251037; doi:10.3389/fimmu.2020.00844)
Supplement: Supplementary file 1 [file Data_Sheet_1.pdf]

Supplementary Materials

Role of Small Intestine and Gut Microbiome in Plant-based Oral Tolerance for Hemophilia

Sandeep RP Kumar[1], Xiaomei Wang[2], Nagavardhini Avuthu[3], Thais B. Bertolini[1], Cox Terhorst[4], Chittibabu Guda[3], Henry Daniell[5], Roland W. Herzog[1,2]

[1] Herman B Wells Center for Pediatric Research, IAPUI, Indianapolis, IN, USA.
[2] Department of Pediatrics, University of Florida, Gainesville, FL, USA.
[3] Department of Genetics, Cell Biology and Anatomy, University of Nebraska Medical Center, Omaha, NE, USA
[4] Division of Immunology, Beth Israel Deaconess Medical Center (BIDMC), Harvard Medical School, Boston, MA, USA.
[5] Department of Basic and Translational Sciences, School of Dental Medicine, University of Pennsylvania, Philadelphia, PA 19104, USA.

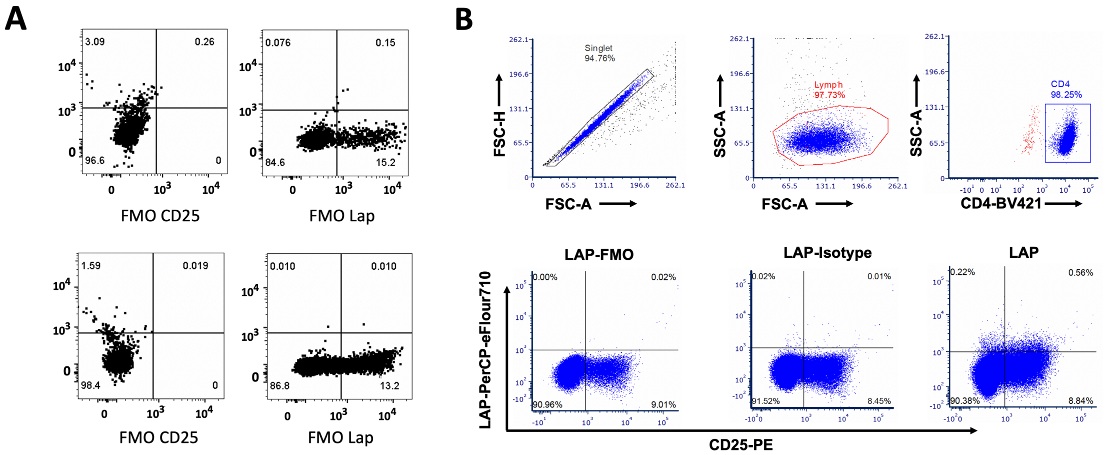

Supplementary Figure S1: Controls for LAP stains. **A.** Representative dot plots showing FMO for CD25 and LAP stains of LAPs (lamina propria lymphocytes) isolated from orally tolerized mice. **B.** Representative dot plots showing gating scheme for LAP^{+} cells staining for splenocytes. FMO and isotype controls were used to demonstrate LAP-specific staining.

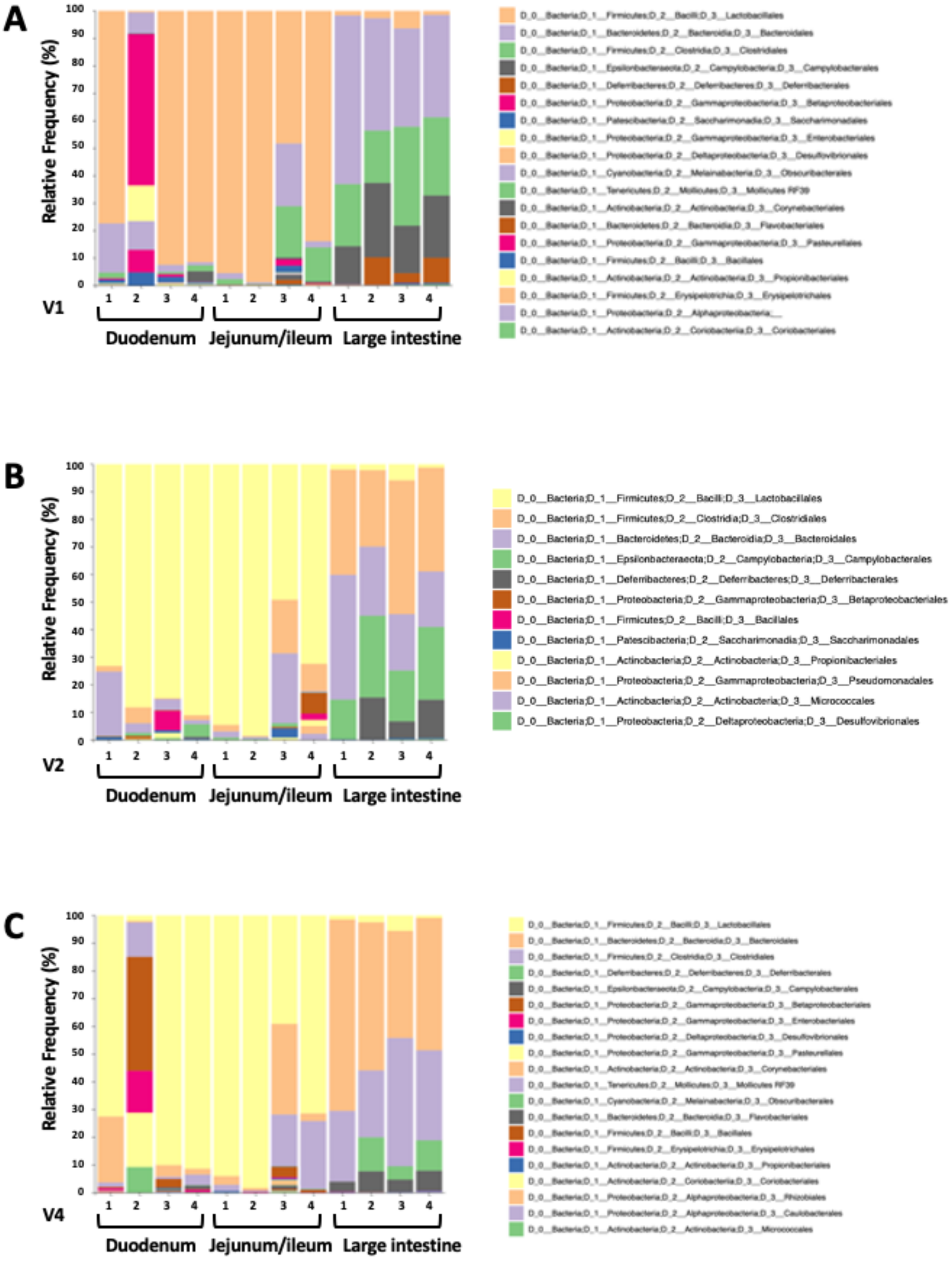
A
Relative Frequency (%)
100
90
80
70
60
50
40
30
20
10
0
V1
1 2 3 4 1 2 3 4 1 2 3 4
Duodenum Jejunum/ileum Large intestine
D_0__Bacteria;D_1__Firmicutes;D_2__Bacilli;D_3__Lactobacillales
D_0__Bacteria;D_1__Bacteroidetes;D_2__Bacteroidia;D_3__Bacteroidales
D_0__Bacteria;D_1__Firmicutes;D_2__Clostridia;D_3__Clostridiales
D_0__Bacteria;D_1__Epsilonbacteraeota;D_2__Campylobacteria;D_3__Campylobacterales
D_0__Bacteria;D_1__Deferribacteres;D_2__Deferribacteres;D_3__Deferribacterales
D_0__Bacteria;D_1__Proteobacteria;D_2__Gammaproteobacteria;D_3__Betaproteobacteriales
D_0__Bacteria;D_1__Patescibacteria;D_2__Saccharimonadia;D_3__Saccharimonadales
D_0__Bacteria;D_1__Proteobacteria;D_2__Gammaproteobacteria;D_3__Enterobacteriales
D_0__Bacteria;D_1__Proteobacteria;D_2__Deltaproteobacteria;D_3__Desulfovibrionales
D_0__Bacteria;D_1__Cyanobacteria;D_2__Melainabacteria;D_3__Obscuribacterales
D_0__Bacteria;D_1__Tenericutes;D_2__Mollicutes;D_3__Mollicutes RF39
D_0__Bacteria;D_1__Actinobacteria;D_2__Actinobacteria;D_3__Corynebacteriales
D_0__Bacteria;D_1__Bacteroidetes;D_2__Bacteroidia;D_3__Flavobacteriales
D_0__Bacteria;D_1__Proteobacteria;D_2__Gammaproteobacteria;D_3__Pasteurellales
D_0__Bacteria;D_1__Firmicutes;D_2__Bacilli;D_3__Bacillales
D_0__Bacteria;D_1__Actinobacteria;D_2__Actinobacteria;D_3__Propionibacteriales
D_0__Bacteria;D_1__Firmicutes;D_2__Erysipelotrichia;D_3__Erysipelotrichales
D_0__Bacteria;D_1__Proteobacteria;D_2__Alphaproteobacteria;__
D_0__Bacteria;D_1__Actinobacteria;D_2__Coriobacteriia;D_3__Coriobacteriales
B
Relative Frequency (%)
100
90
80
70
60
50
40
30
20
10
0
V2
1 2 3 4 1 2 3 4 1 2 3 4
Duodenum Jejunum/ileum Large intestine
D_0__Bacteria;D_1__Firmicutes;D_2__Bacilli;D_3__Lactobacillales
D_0__Bacteria;D_1__Firmicutes;D_2__Clostridia;D_3__Clostridiales
D_0__Bacteria;D_1__Bacteroidetes;D_2__Bacteroidia;D_3__Bacteroidales
D_0__Bacteria;D_1__Epsilonbacteraeota;D_2__Campylobacteria;D_3__Campylobacterales
D_0__Bacteria;D_1__Deferribacteres;D_2__Deferribacteres;D_3__Deferribacterales
D_0__Bacteria;D_1__Proteobacteria;D_2__Gammaproteobacteria;D_3__Betaproteobacteriales
D_0__Bacteria;D_1__Firmicutes;D_2__Bacilli;D_3__Bacillales
D_0__Bacteria;D_1__Patescibacteria;D_2__Saccharimonadia;D_3__Saccharimonadales
D_0__Bacteria;D_1__Actinobacteria;D_2__Actinobacteria;D_3__Propionibacteriales
D_0__Bacteria;D_1__Proteobacteria;D_2__Gammaproteobacteria;D_3__Pseudomonadales
D_0__Bacteria;D_1__Actinobacteria;D_2__Actinobacteria;D_3__Micrococcales
D_0__Bacteria;D_1__Proteobacteria;D_2__Deltaproteobacteria;D_3__Desulfovibrionales
C
Relative Frequency (%)
100
90
80
70
60
50
40
30
20
10
0
V4
1 2 3 4 1 2 3 4 1 2 3 4
Duodenum Jejunum/ileum Large intestine
D_0__Bacteria;D_1__Firmicutes;D_2__Bacilli;D_3__Lactobacillales
D_0__Bacteria;D_1__Bacteroidetes;D_2__Bacteroidia;D_3__Bacteroidales
D_0__Bacteria;D_1__Firmicutes;D_2__Clostridia;D_3__Clostridiales
D_0__Bacteria;D_1__Deferribacteres;D_2__Deferribacteres;D_3__Deferribacterales
D_0__Bacteria;D_1__Epsilonbacteraeota;D_2__Campylobacteria;D_3__Campylobacterales
D_0__Bacteria;D_1__Proteobacteria;D_2__Gammaproteobacteria;D_3__Betaproteobacteriales
D_0__Bacteria;D_1__Proteobacteria;D_2__Gammaproteobacteria;D_3__Enterobacteriales
D_0__Bacteria;D_1__Proteobacteria;D_2__Deltaproteobacteria;D_3__Desulfovibrionales
D_0__Bacteria;D_1__Proteobacteria;D_2__Gammaproteobacteria;D_3__Pasteurellales
D_0__Bacteria;D_1__Actinobacteria;D_2__Actinobacteria;D_3__Corynebacteriales
D_0__Bacteria;D_1__Tenericutes;D_2__Mollicutes;D_3__Mollicutes RF39
D_0__Bacteria;D_1__Cyanobacteria;D_2__Melainabacteria;D_3__Obscuribacterales
D_0__Bacteria;D_1__Bacteroidetes;D_2__Bacteroidia;D_3__Flavobacteriales
D_0__Bacteria;D_1__Firmicutes;D_2__Bacilli;D_3__Bacillales
D_0__Bacteria;D_1__Firmicutes;D_2__Erysipelotrichia;D_3__Erysipelotrichales
D_0__Bacteria;D_1__Actinobacteria;D_2__Actinobacteria;D_3__Propionibacteriales
D_0__Bacteria;D_1__Actinobacteria;D_2__Coriobacteriia;D_3__Coriobacteriales
D_0__Bacteria;D_1__Proteobacteria;D_2__Alphaproteobacteria;D_3__Rhizobiales
D_0__Bacteria;D_1__Proteobacteria;D_2__Alphaproteobacteria;D_3__Caulobacterales
D_0__Bacteria;D_1__Actinobacteria;D_2__Actinobacteria;D_3__Micrococcales

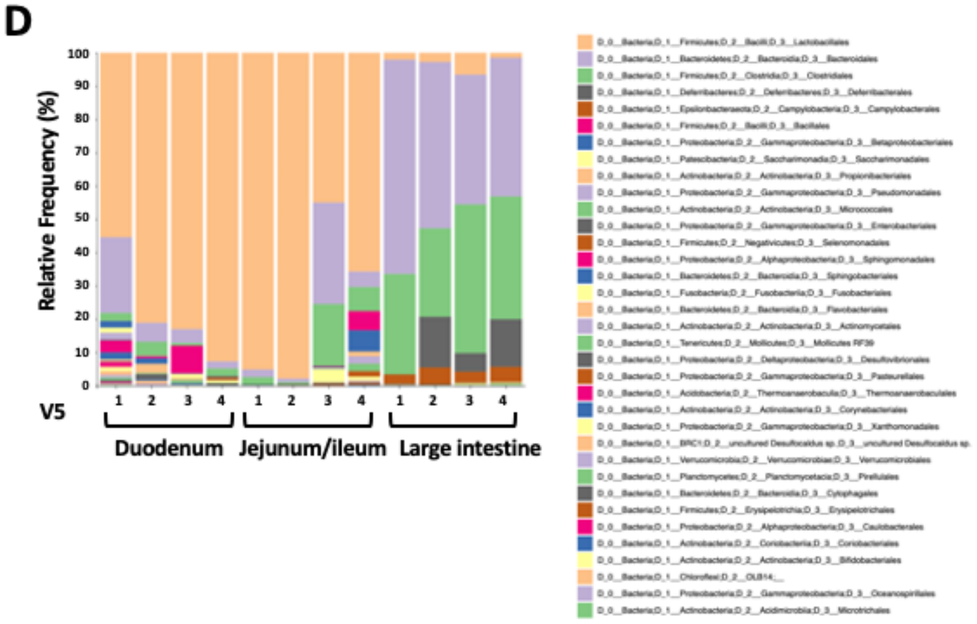

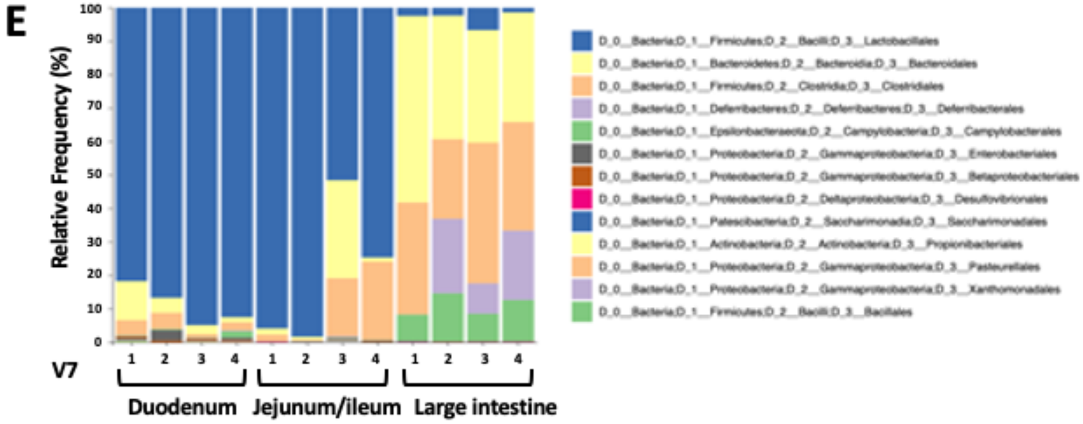

Supplementary Figure S2. Relative frequencies of bacterial orders in duodenum, jejunum/ileum, and small intestine of hemophilia B mice (n=4) as determined by bioinformatic analysis of NGS data. Data were obtained from amplification of variable region v1v2 (**A**), variable region v2v3 (**B**), variable region v4v5 (**C**), variable region v5v7 (**D**), and variable region v7v9 (**E**).

A

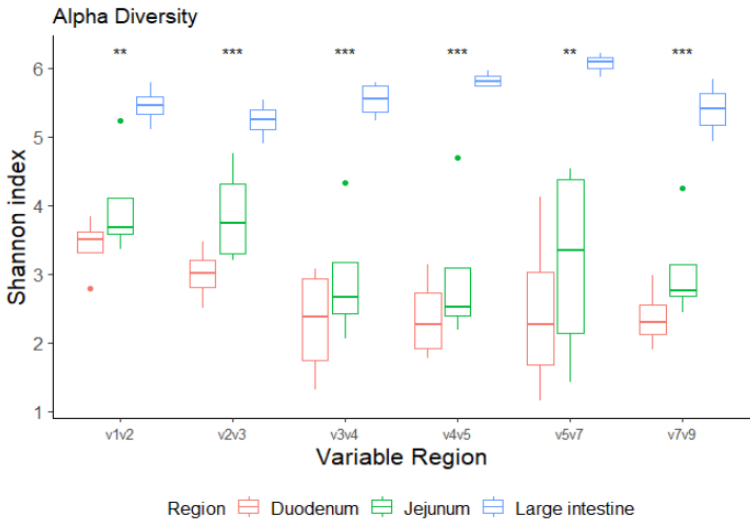

B

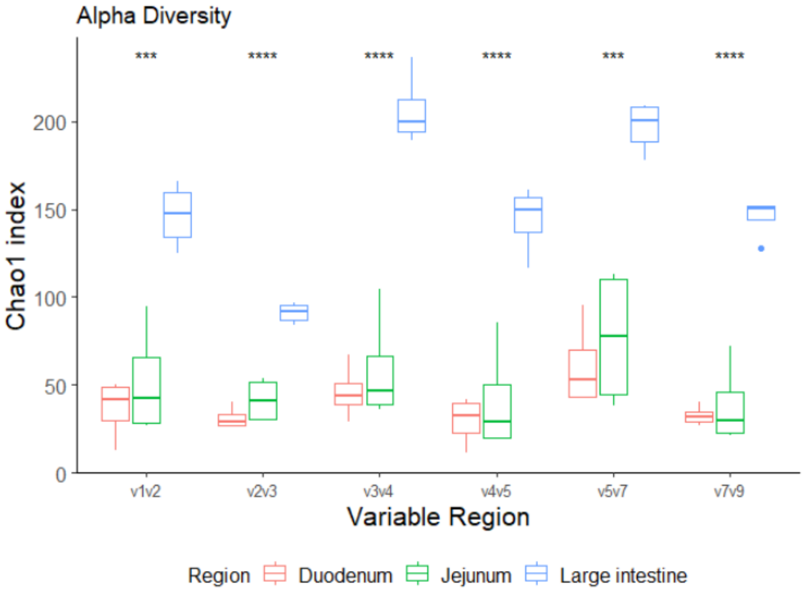

Supplementary Figure S3: Variations in alpha diversity of hemophilia B mice gut microbiome. (A) Box plots showing Shannon Index variation, and (B) Box plots showing Chao1 richness estimator variation across the gut regions in 16S rRNA amplified variable regions (data rarefied to the minimum sampling depth in each 16S rRNA variable regions). Statistical test ANOVA, i.e., `****` ≤ 1e-04, `***` ≤ 0.001, `**` ≤ 0.01.

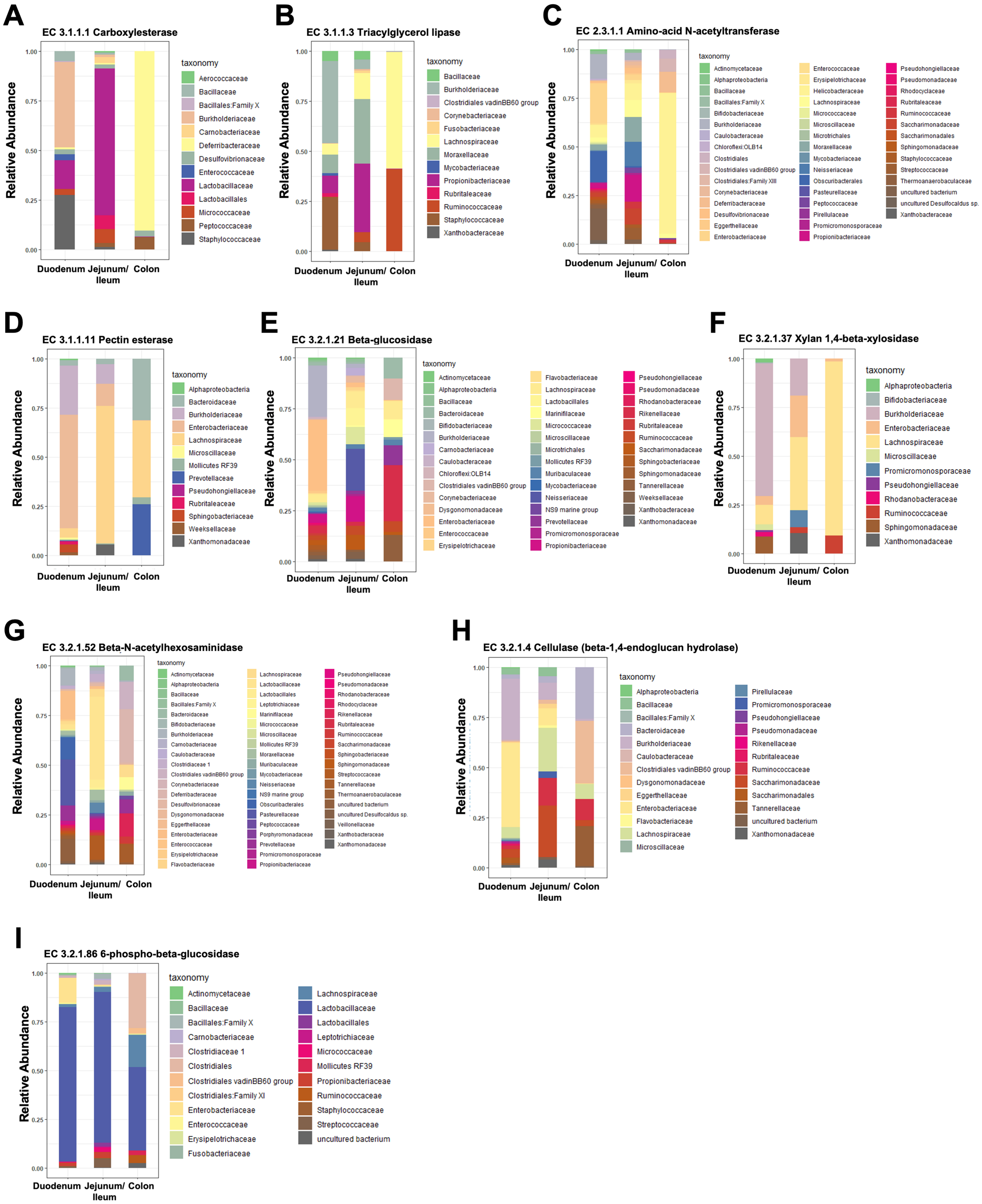

Supplementary Figure S4: Relative abundance of bacterial families producing enzymes that degrade plant cell wall components were identified in the duodenum, jejunum and large intestine of hemophilia B mice. Results are shown as highest abundance of each enzyme from the tested amplicon regions in duodenum, jejunum/ileum, and large

intestine of the hemophilia B mice. **A.** Carboxylesterase. **B.** Triacylglycerol lipase. **C.** Amino-acid N-acetyltransferase. **D.** Pectinesterase. **E.** β-glucosidase. **F.** Xylan 1,4-β-xylosidase. **G.** β-N-acetylhexosaminidase. **H.** Cellulase (-1,4-endoglucan hydrolase). **I.** 6-phospho-β-glucosidase.

Enzyme	Region	Best Amplicon	Rep-1	Rep-2	Rep-3	Rep-4	Median	Mean
EC:3.1.1.3	Duodenum	v1v2	0.0015	0.0523	0.0030	0.0009	0.0022	0.0144
EC:3.1.1.3	Jej./Ileum	v3v4	0.0000	0.0003	0.0109	0.0012	0.0007	0.0031
EC:3.1.1.3	Colon	v2v3	0.0000	0.0000	0.0000	0.0000	0.0000	0.0000
EC:3.1.1.1	Duodenum	v5v7	0.0014	0.0044	0.0321	0.0015	0.0030	0.0099
EC:3.1.1.1	Jej./Ileum	v7v9	0.0280	0.0885	0.0009	0.0005	0.0145	0.0295
EC:3.1.1.1	Colon	v7v9	0.0004	0.0248	0.0090	0.0221	0.0156	0.0141
EC:3.2.1.86	Duodenum	v3v4	0.9649	0.9015	1.0682	0.9971	0.9810	0.9829
EC:3.2.1.86	Jej./Ileum	v3v4	0.9181	0.4889	0.7369	0.8171	0.7770	0.7402
EC:3.2.1.86	Colon	v3v4	0.1059	0.1128	0.2286	0.1393	0.1260	0.1466
EC:3.2.1.52	Duodenum	v1v2	0.0573	0.1458	0.0202	0.0095	0.0388	0.0582
EC:3.2.1.52	Jej./Ileum	v5v7	0.0061	0.0335	0.1161	0.1687	0.0748	0.0811
EC:3.2.1.52	Colon	v4v5	0.4084	0.3356	0.3149	0.3271	0.3314	0.3465
EC:3.2.1.4	Duodenum	v1v2	0.0066	0.1224	0.0060	0.0036	0.0063	0.0347
EC:3.2.1.4	Jej./Ileum	v4v5	0.0000	0.0004	0.0404	0.0083	0.0043	0.0123
EC:3.2.1.4	Colon	v2v3	0.0747	0.0596	0.0579	0.0434	0.0587	0.0589
EC:2.3.1.1	Duodenum	v1v2	0.0085	0.1213	0.0094	0.0197	0.0145	0.0397
EC:2.3.1.1	Jej./Ileum	v2v3	0.0036	0.0017	0.0519	0.0599	0.0277	0.0293
EC:2.3.1.1	Colon	v2v3	0.1376	0.2199	0.1716	0.2101	0.1909	0.1848
EC:3.2.1.21	Duodenum	v5v7	0.1607	0.0754	0.0267	0.0244	0.0511	0.0718
EC:3.2.1.21	Jej./Ileum	v4v5	0.0116	0.0059	0.2721	0.2014	0.1065	0.1227
EC:3.2.1.21	Colon	v4v5	0.6228	0.4700	0.5436	0.4965	0.5201	0.5332
EC:3.2.1.37	Duodenum	v1v2	0.0017	0.0660	0.0001	0.0008	0.0013	0.0172
EC:3.2.1.37	Jej./Ileum	v7v9	0.0000	0.0001	0.0143	0.0005	0.0003	0.0037
EC:3.2.1.37	Colon	v3v4	0.0092	0.0085	0.0167	0.0138	0.0115	0.0120
EC:3.2.1.78	Duodenum	v5v7	0.0222	0.0037	0.0032	0.0009	0.0034	0.0075
EC:3.2.1.78	Jej./Ileum	v4v5	0.0015	0.0005	0.0189	0.0020	0.0018	0.0057
EC:3.2.1.78	Colon	v1v2	0.0733	0.0388	0.0336	0.0363	0.0375	0.0455
EC:3.1.1.11	Duodenum	v1v2	0.0017	0.0266	0.0000	0.0010	0.0013	0.0073
EC:3.1.1.11	Jej./Ileum	v3v4	0.0001	0.0003	0.0074	0.0009	0.0006	0.0022
EC:3.1.1.11	Colon	v3v4	0.0525	0.0314	0.0222	0.0262	0.0288	0.0331
EC:3.2.1.8	Duodenum	v3v4	0.0000	0.0000	0.0001	0.0002	0.0000	0.0001
EC:3.2.1.8	Jej./Ileum	v3v4	0.0000	0.0000	0.0000	0.0000	0.0000	0.0000
EC:3.2.1.8	Colon	v5v7	0.0162	0.0100	0.0119	0.0105	0.0112	0.0121

Supplemetary Table S1. Species relative frequencies of bacteria producing the following enzymes in duodenum, jejunum/ileum, and large intestine of hemophilia B mice as predicted by PICRUSt2 on different 16S rRNA variable region taxonomic profiles.
